# Gut Fungal Microbiota Alterations in Pulmonary Arterial Hypertensive Rats

**DOI:** 10.3390/biomedicines12020298

**Published:** 2024-01-27

**Authors:** Yihang Chen, Liukun Meng, Wen Yuan, Zehan Gao, Xun Zhang, Boqia Xie, Jiawei Song, Jifeng Li, Jiuchang Zhong, Xiaoyan Liu

**Affiliations:** 1 Department of Cardiology, Beijing Chao-Yang Hospital, Capital Medical University, Beijing 100020, China; yihangchen15@mail.ccmu.edu.cn (Y.C.); dr.boqiaxie@mail.ccmu.edu.cn (B.X.); songjiawei27@163.com (J.S.); 2 Heart Center and Beijing Key Laboratory of Hypertension, Beijing Institute of Respiratory Medicine, Beijing Chao-Yang Hospital, Capital Medical University, Beijing 100020, China; 3 State Key Laboratory of Cardiovascular Disease, Fuwai Hospital, National Center for Cardiovascular Disease, Peking Union Medical College, Chinese Academy of Medical Sciences, Beijing 100032, China; mengliukun@fuwaihospital.org; 4 Medical Research Center, Beijing Institute of Respiratory Medicine and Beijing Chao-Yang Hospital, Capital Medical University, Beijing 100020, China; rainbow@ccmu.edu.cn; 5 Department of Respiratory and Critical Care Medicine, Beijing Institute of Respiratory Medicine and Beijing Chao-Yang Hospital, Capital Medical University, Beijing 100020, China; gzhhh1009@163.com (Z.G.); lijifeng@ccmu.edu.cn (J.L.); 6 Institute of Microbiology, Chinese Academy of Sciences, Beijing 100101, China; zhangxun@im.ac.cn

**Keywords:** pulmonary arterial hypertension, mycobiome, fungi, microbiome, internal transcribed spacer 1

## Abstract

The gut microbiome’s imbalance has been implicated in the pathogenesis of pulmonary arterial hypertension (PAH), yet the contribution of the gut mycobiome remains largely unclear. This study delineates the gut mycobiome profile in PAH and examines its interplay with the bacterial microbiome alterations. Fecal samples from monocrotaline-induced PAH rats and matched controls were subjected to internal transcribed spacer 1 (ITS1) sequencing for fungal community assessment and 16S ribosomal RNA (rRNA) gene sequencing for bacterial community characterization. Comparative analysis revealed no significant disparities in the overall mycobiome diversity between the PAH and control groups. However, taxonomic profiling identified differential mycobiome compositions, with the PAH group exhibiting a significant enrichment of genera such as *Wallemia, unidentified_Branch02, Postia, Malassezia, Epicoccum, Cercospora,* and *Alternaria.* Conversely, genera *Xeromyces, unidentified_Plectosphaerellaceae*, and *Monilia* were more abundant in the controls. Correlations of *Malassezia* and *Wallemia* abundance with hemodynamic parameters were observed. Indications of bidirectional fungal–bacterial community interactions were also noted. This investigation reveals distinct gut mycobiome alterations in PAH, which are intricately associated with concurrent bacterial microbiome changes, suggesting a possible contributory role of gut fungi in PAH pathophysiology. These findings underscore the potential for novel gut mycobiome-targeted therapeutic interventions in PAH management.

## 1. Introduction

Pulmonary arterial hypertension (PAH) is a complex and progressive condition characterized by elevated blood pressure in the pulmonary arteries, leading to right-sided heart failure and premature death. The gut microbiota, plays a crucial role in PAH, includes bacteria, fungi, viruses, and other microorganisms residing in the gastrointestinal tract. Previous studies [1,2,3] have extensively documented the relationship between gut bacterial dysbiosis and PAH, highlighting the interaction between the gut microbiota and cardiovascular health. Dysbiosis, an imbalance in the gut microbiome, is associated with various cardiovascular disorders. However, despite the extensive research on the bacterial aspect of the intestinal microbiome, the significance of gut fungi, also known as the gut mycobiota, in relation to PAH remains largely unexplored.

Although fungi make up only 0.1–1% of the human gut microbiota, which exert a critical role in maintaining intestinal equilibrium and overall wellness [4], and possess the capacity to modulate bacterial populations, fostering an interactive and dynamic relationship with the host’s immune system [5]. Certain fungi are even regarded as potential probiotics, offering preventative or therapeutic benefits for various diseases. For example, *Saccharomyces boulardii* exerts anti-pathogenic properties by upholding cellular physiology, obstructing the adherence of pathogens and their toxins, and engaging with the normal gut flora or aiding in the replenishment of short-chain fatty acid concentrations within the gut [6]. Furthermore, mono-colonization with *Saccharomyces cerevisiae* can alleviate intestinal ischemia-reperfusion injury by enhancing tight junction barrier function, reducing mucosal damage and epithelial permeability, and decreasing the release of inflammatory cytokines [7]. Nevertheless, should the fine balance between the immune system and symbiotic fungi be disturbed, the fungi may turn pathogenic, contributing to an array of disorders that include inflammatory bowel disease (IBD), hepatocellular carcinoma, and hypertension [8,9,10]. Specific fungal pathogens have the ability to induce the synthesis of cytokines and chemokines from phagocytes, as well as activate the NOD-like receptor family pyrin domain containing 3 (NLRP3) inflammasome through different receptors and signaling pathways [11].

The relationship between the gut mycobiome and PAH remains a significant research gap, with most studies focusing on bacterial dysbiosis and neglecting the role of fungi in PAH. This lack of understanding limits the potential for comprehensive therapeutic approaches that consider the entire gut microbiome, including both bacterial and fungal components. Addressing this gap is crucial for developing more effective strategies for managing and treating PAH.

This present research aims to bridge this gap by characterizing the gut mycobiome profile in monocrotaline (MCT)-induced PAH rats, which is the predominant experimental paradigm for studying PAH in rodents. Techniques such as internal transcribed spacer 1 (ITS1) and 16S rRNA gene sequencing were utilized to analyze fecal samples from both MCT-induced PAH and control rats, focusing on examining the diversity and composition of the gut mycobiome and its correlation with changes in the bacterial microbiome in PAH. By exploring these associations, this work seeks to uncover potential pathophysiological roles of gut fungi in PAH and their interaction with gut bacteria, thereby offering new insights into pathogenesis and potential therapeutic targets for PAH.

## 2. Materials and Methods

### 2.1. Animals and Groupings

Fourteen male Sprague Dawley (SD) rats, aged five weeks and weighing between 180and 210 g, were obtained from Beijing Vital River Laboratory Animal Technology Co., Ltd. Beijing, China. SD rats were housed in a controlled environment free from specific pathogens (SPF) at Beijing Chaoyang Hospital. Following a week of adaptive nourishment within a controlled animal enclosure, PAH was triggered through intraperitoneal administration of MCT at a dosage of 60 mg/kg (provided by Sigma-Aldrich Co. LLC, St. Louis, MO, USA). The MCT powder was mixed with a solution containing ethanol and saline, with a ratio of 2 to 8. The rats were divided into two groups, namely the control (CON) group that received the solvent injection (n = 7), and the MCT-PAH group that received the MCT injection (n = 7), in a random manner. Four weeks after MCT injection, echocardiography was performed. Subsequently, the rats were anesthetized by pentobarbital sodium (40 mg/kg) and sacrificed for hemodynamic measurements.

This research was carried out in accordance with the ethical standards set by the Animal Experimentation Ethics Committee of Capital Medical University, Beijing, China (AEEI-2022-011), and followed the current National Institutes of Health (NIH) guidelines for animal welfare and experimental protocols and conducted following ethical guidelines approved by the Animal Experimentation Ethics Committee of Capital Medical University, Beijing, China (AEEI-2022-011), and adhered to current NIH guidelines for animal care and experimental procedures.

### 2.2. Echocardiography

Echocardiography assessment was conducted four weeks after MCT administration, following the previously outlined procedure [12]. An experienced operator performed the echocardiography using a Visual Sonic VEVO 2100. To assess the function of the right ventricle (RV), echocardiography was used to evaluate tricuspid annular plane systolic excursion (TAPSE), right ventricular anterior wall (RVAW), right ventricular internal dimension (RVID), and pulmonary artery acceleration time (PAAT). Data was collected from 10 successive heartbeats and utilized to normalize variations between beats.

### 2.3. Hemodynamic Measurements and Tissue Processing

After the echocardiography evaluation, both the CON and MCT-PAH groups of rats underwent the right heart catheterization (RHC) technique in order to gauge the right ventricular systolic pressure (RVSP). Afterwards, the lungs and hearts were immediately extracted and measured in terms of weight. We measured the weights of the right ventricular free wall (RV) and the left ventricle (LV) plus septum (S) when they were dry, and then calculated the RV/LV + S ratio. These measurements provide valuable information about the severity of pulmonary hypertension and the resulting changes in the structure and function of the heart in the MCT-induced PAH rat model.

### 2.4. Histological Examination

After being immersed in 10% neutral buffer formalin for a duration of 24 h, the lung tissues were subsequently encased in paraffin and sliced into sections measuring 4 μm in thickness. Vascular remodeling was assessed by staining cross-sections with Elastica van Giesson (EVG) and hematoxylin and eosin (HE). EVG staining was utilized to measure the pulmonary artery media thickness. Each lung section was examined and all vessels with a diameter smaller than 100 μm were tallied. These vessels were then classified according to their level of muscularization, which included fully muscularized (having two distinct and continuous elastic lamina), partially muscularized (with the second elastic lamina not continuous [<50%]), or non-muscularized (possessing a single elastic lamina). At least 40 ships were tallied per section, and the ratio of ships in every classification was represented as a percentage of the overall ships tallied. Morphometric analysis was performed by two independent blinded examiners, with inter-person variability of <10%. The level of blockage was computed in pulmonary vessels with external sizes varying from 25 to 100 μm utilizing the subsequent equation: (area of outer vessel area of inner vessel) divided by area of outer vessel. An observer who was unaware of the experimental groups measured and averaged the values of over 10 randomly chosen vessels for each animal, which were typically round or oval in shape.

### 2.5. Sample Acquisition and DNA Extraction

After the completion of the 4-week experiment, we collected stool samples from each rat in sterile containers, promptly freezing them at −80 °C for further analysis. The genomic DNA was extracted from the fecal samples at Oriental Yeekang (Beijing, China) Medicine Technology Co., Ltd., using a Qiagen kit (located in Valencia, CA, USA), following the instructions provided by the manufacturer. Electrophoresis was performed on a 1.0% agarose gel supplemented with 0.5 mg/mL ethidium bromide to verify the quality and quantity of the isolated DNA. For the analysis of the fungal microbiome, we amplified the fungal internal transcribed spacer 1 (ITS1) gene using bar-coded primers specific to ITS1: ITS1F-F (5′-CTTGGTCATTTAGAGGAAGTAA-3′) and ITS1F-R (5′-GCTGCGTTCTTCATCGATGC-3′). Furthermore, we magnified the V3–V4 segments of the 16S rRNA molecule to conduct an analysis of the bacterial microbiome. PCR reactions were performed with the Phusion High-Fidelity PCR Master Mix, utilizing around 50 ng of extracted DNA per reaction. The thermocycling parameters included an initial denaturation phase at 98 °C for 15 s, followed by 30 cycles of denaturation at 98 °C for 15 s, annealing at 58 °C for 15 s, extension at 72 °C for 15 s, and a final extension step at 72 °C for 1 min. After purification, the PCR products were quantified using the Universal DNA Purification Kit (TianGen, China, Catalog # DP214). Based on fluorescence quantitative results and sequencing quantity requirements, we combined the samples in appropriate proportions. Afterwards, we utilized the NEB Next Ultra DNA Library Prep Kit (New England Biolabs, Ipswich, MA, USA) for the creation of sequencing libraries. Finally, sequencing was performed on a NovaSeq 6000 platform (Illumina, San Diego, CA, USA) using a 2 × 150 bp sequencing strategy.

### 2.6. Bioinformatics Analysis and Data Analysis

The original high-throughput sequencing data were demultiplexed using QIIME v1.9.1. In order to guarantee dependable outcomes in subsequent bioinformatics investigations, QIIME v1.9.1 was employed for the exclusion of sequences with a length of less than 200 bp and the elimination of chimera. Sequences of excellent quality were grouped into operational taxonomic units (OTUs) that were defined based on a 97% similarity threshold. The representative sequence for each OTU was chosen as the most abundant sequence. The BLAST algorithm was utilized to taxonomically classify the OTUs by searching the representative sequences set against the UNITE (v8.2) (https//unite.ut.ee, accessed on 12 December 2023) database. The vegan software, specifically the R version 4.2.2, was utilized for the computation of alpha and beta diversity. Gut microbial alpha diversity was characterized by calculating the Chao1, Shannon, and Simpson diversity indexes according to the relative abundance distribution of OTUs in each sample. Differences and similarities among various samples and groups were assessed by analyzing beta diversity through non-metric multidimensional scaling (NMDS) and principal coordinate analysis (PCoA) utilizing Bray–Curtis distance. The Wilcoxon test was used to perform alpha diversity and differential abundance analysis between the CON and MCT-PAH groups, with *p*-values being adjusted using the Benjamini–Hochberg algorithm. To assess the statistical significance of beta-diversity clustering, PERMANOVA and ANOSIM analyses were utilized. The edgeR algorithm was utilized to perform differential abundance analysis. We used Spearman’s method with R version 4.2.2 to establish the associations between hemodynamics and fungal genera in fecal samples. Spearman’s rank correlation was used to analyze the correlation between bacteria and fungi at the genus level across different kingdoms. Only correlations with a *p*-value below 0.05 were reported, and the heatmap software was utilized to generate the correlation heatmap.

### 2.7. Statistical Analysis

GraphPad Prism 9.1 software was utilized for conducting data analysis and generating graphs. The student’s t-test was used for normalized continuous variables, whereas the Wilcoxon rank-sum test was employed for non-normal continuous variables. The analysis and generation of figures for alpha diversity, beta diversity, and differential abundance of all features were performed using R (Version 4.2.2) with the vegan and ggpubr packages, as well as custom R scripts. The Wilcoxon test was utilized to analyze alpha diversity and differential abundance between the CON and MCT-PAH groups, with *p*-values adjusted using the Benjamini–Hochberg algorithm. The Spearman method was employed for correlation analyses. Spearman’s rank correlation was utilized to perform inter-kingdom correlation analysis, specifically examining the relationship between bacteria and fungi at the genus level. Reported are only correlations that have a *p*-value below 0.05. The means ± SD were presented as the results, and *p*-values that were less than 0.05 were regarded as statistically significant.

## 3. Results

### 3.1. Severe PAH Developed in Monocrotaline (MCT)-Induced Rats

**Figure 1:**
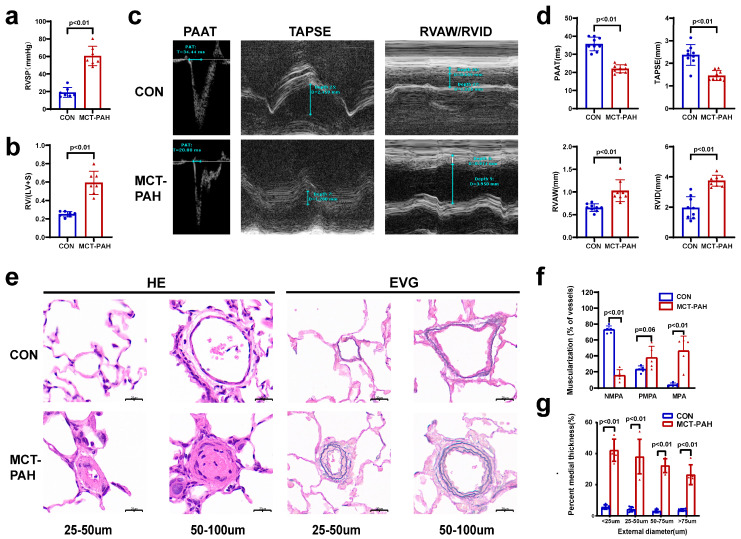
Monocrotaline (MCT)-induced rats developed severe pulmonary arterial hypertension. (**a**,**b**) Sprague Dawley rats demonstrated increased right ventricular systolic pressure (RVSP) (**a**) and a higher right ventricular hypertrophy index (RVHI), which was calculated by RV/(LV + S) ratio (**b**) (n = 7 for each group). Data are presented as mean ± s.e.m. (**c**) Echocardiographic measurements (PAAT, TAPSE, RVID, and RVAW) and images. (**d**) Echocardiography was conducted on CON and MCT rats on Day 28 post-MCT injection to assess PAAT, TAPSE, RVID, and RVAW (n = 7). (**e**) Representative HE- and EVG-stained images of pulmonary arteries (PAs) with diameters of 25–50 μm or 51–100 μm. Scale bar represents 20 μm. (**f**) Proportion of non-muscularized (NMPA), partially muscularized (PMPA), and fully muscularized (MPA) pulmonary arterioles in MCT-induced rats (n = 5 rats per group, forty PAs per rat). (**g**) Quantification of vascular medial thickness in diameters of <25 μm, 25–50 μm, 50–75 μm, or 75–100 μm from EVG-stained images (n = 5); one-way ANOVA with Tukey’s post-hoc test. Data are presented as mean ± s.e.m. TAPSE: tricuspid annular plane systolic excursion; RV/(LV + S): the weight of the right ventricle (RV)/the combined weight of the left ventricle (LV) and septum (S); RVAW: right ventricular anterior wall; RVID: right ventricular internal dimension; PAAT: pulmonary artery acceleration time; EVG: Elastica van Gieson staining; HE: hematoxylin and eosin.

Compared to the control rats, the MCT-induced PAH rats exihibited a significant increase in the right ventricular systolic pressure (RVSP) (Figure 1a) and right ventricular hypertrophy index (RVHI) (Figure 1b). The RVHI is calculated by dividing the weight of the right ventricle (RV) by the combined weight of the left ventricle (LV) and septum (RV/(LV + S) ratio). An increased RVHI indicates an abnormal growth of the right ventricle, which is often associated with PAH. Furthermore, echocardiography demonstrated that MCT administration resulted in a notable reduction in pulmonary artery acceleration time (PAAT) and tricuspid annular plane systolic excursion (TAPSE) in rats with PAH, accompanied by a significant rise in the right ventricular anterior wall (RVAW) thickness and right ventricular internal diameter (RVID) (Figure 1c,d). To assess the pulmonary vascular remodeling, hematoxylin and eosin (HE) and Elastica van Gieson (EVG) staining of the intermediate pulmonary vessels (25–100 μm) was performed. These stains revealed muscle thickening and increased medial thickness in distal pulmonary arterioles following MCT treatment(Figure 1e–g). These findings indicate the successful establishment of PAH in the MCT-treated group.

### 3.2. Characteristics of the Sequence Datasets

To analyze the fecal fungal community in PAH, we conducted internal transcribed spacer 1 (ITS1) region sequencing in MCT-induced PAH rats. A total of 1,184,755 original raw reads were obtained from the 14 fecal samples, with an average of 84,625 reads per sample. The sequences were grouped into 2565 operational taxonomic units (OTUs) with 97% nucleotide sequence similarity (Table 1). The number of sequences that were assignable to known taxa in the database gradually decreased from phylum to species. In total, we detected 12 phyla, 36 classes, 88 orders, 186 families, 316 genera, and 414 species of fungi in the fecal samples.

### 3.3. Ecological Features of the Fecal Fungal Flora in PAH Rats

**Figure 2:**
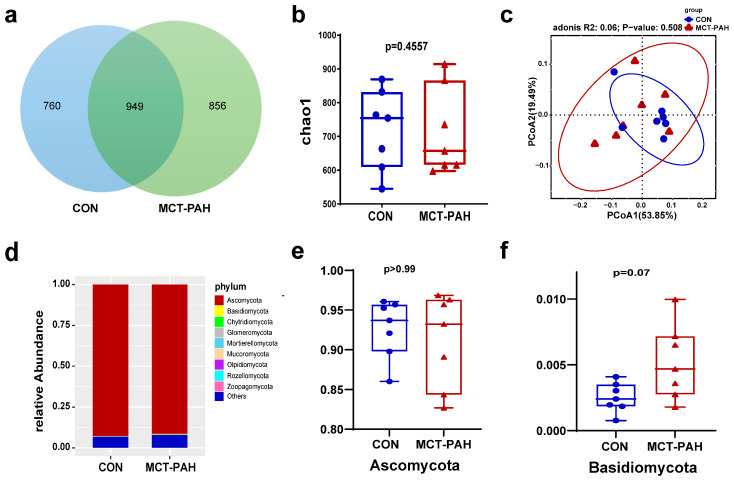
Ecological features of the fecal fungal flora in PAH rats. (**a**) Venn diagrams for gut fungi OTU distribution. Statistical results of the alpha diversity between the two groups. (**b**) Chao1 index. (**c**) Principal coordinates analysis (PCoA) diagram based on Bray–Curtis dissimilarity. (**d**) ANOSIM analysis based on Bray–Curtis dissimilarity. (**e**) The Ascomycota relative abundance of CON and MCT-PAH groups. (**f**) The Basidiomycota relative abundance of CON and MCT-PAH groups. Statistical significance was assessed by PERMANOVA test. MCT: monocrotaline. PAH: pulmonary arterial hypertension.

A Venn diagram was utilized to visually represent the shared and unique OTUs between the CON and MCT-PAH groups (Figure 2a). A total of 949 core OTUs, accounting for approximately 37% of the total OTUs, were identified as shared between both groups. Furthermore, 760 OTUs were exclusive to the CON group, while 856 OTUs were specific to the MCT-PAH group.

To further investigate the changes in gut fungal communities in PAH rats, alpha-diversity indices at the OTU level were employed. Fungal alpha diversity was assessed using species abundance (Chao1) (Figure 2b). However, no significant differences were observed between the CON and MCT-PAH groups, although there was an increasing trend in richness among the PAH rats. In order to investigate the beta diversity in fungal composition, a principal coordinates analysis (PCoA) was conducted based on Bray–Curtis dissimilarity at the OTU level (Figure 2c). The PCoA plot did not exhibit a distinct separation between the CON and PAH groups (Adonis, R^2^ = 0.06, *p* = 0.508).

For a structural analysis of the mycobiota, a histogram was generated to highlight the top 10 most abundant phyla (Figure 2d). At the phylum level, both the CON and PAH rats were predominantly populated by *Ascomycota* and *Basidiomycota* (Figure 2d). There was no significant difference in the abundance of *Ascomycota* between the CON and MCT-PAH groups (Figure 2e). However, it was noted that *Basidiomycota* showed a higher abundance in the MCT-PAH group, although this increase did not reach a statistical significance (Figure 2f).

### 3.4. Alterations of Gut Fungal Taxonomic Compositions in PAH

**Figure 3:**
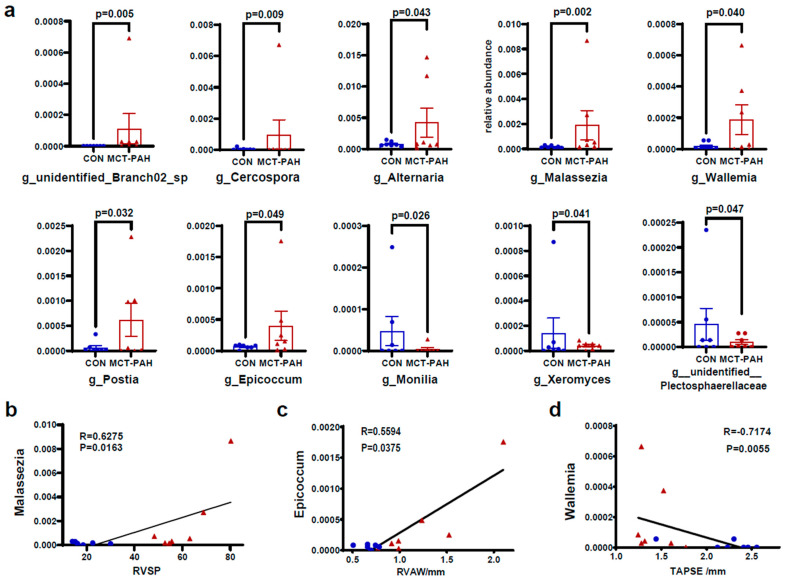
Alterations of gut fungal composition at the genus level in MCT-PAH. (**a**) Differentially enriched genera between CON and MCT-PAH groups. (**b**) Correlation of Malassezia with RVSP. (**c**) Correlation of Epicoccum with TAPSE. (**d**) Correlation of Epicoccum with RVAW. Statistical significance was assessed by edgeR algorithm in R. CON: control; PAH: pulmonary arterial hypertension; RVSP: right ventricular systolic pressure; RVAW: right ventricular anterior wall; TAPSE: tricuspid annular plane systolic excursion; MCT: monocrotaline.

To investigate the changes in fungal taxonomic composition in rats with PAH, we employed the edgeR algorithm in R to analyze data at different classification levels. Our analysis revealed a higher abundance of the genera *unidentified_ Branch02*, *Cercospora, Alternaria, Malassezia, Wallemia, Postia,* and *Epicoccum* in the MCT-PAH group compared to the CON group, while *Monilia, Xeromyces*, and *unidentified_Plectosphaerellaceae* exhibited lower abundance (Figure 3a). Among these genera, *Malassezia* and *Epicoccum* showed the most significant increase in abundance in the MCT-PAH group (Figure 3a).

To explore the potential association between the gut mycobiome and PAH, we conducted an analysis to determine correlations between differentially abundant gut fungal genera and right ventricular function parameters. Our analysis yielded significant findings. Notably, the genus *Malassezia* showed a positive correlation with RVSP (Figure 3b), indicating a potential link between this fungal genus and PAH. Conversely, the fungal genus *Wallemia* exhibited a positive correlation with the right ventricular anterior wall (RVAW) (Figure 3c), while demonstrating a negative correlation with the tricuspid annular plane systolic excursion (TAPSE) (Figure 3d). These correlations offer valuable insights into the potential interactions between specific gut fungal genera and hemodynamic indices in the context of PAH.

**Figure 4:**
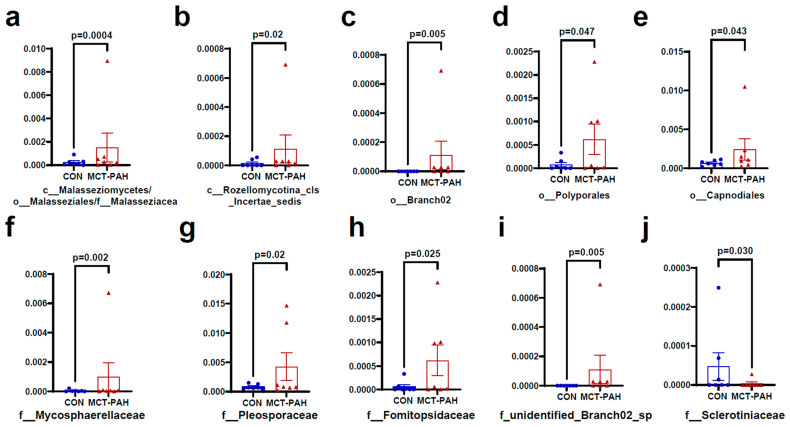
Fungal species differentially enriched in MCT-PAH group at the class, order, and family levels. At the class level, *Malasseziomycetes* (**a**) and *Rozellomycotina_cls_Incertae_sedis* (**b**) were enriched in MCT-PAH group (**b**); in the orders *Malasseziales* (**a**)*, Branch02* (**c**)*, Polyporales* (**d**), and *Capnodiales* (**e**) were enriched in MCT-PAH group. At the family level, *Malasseziaceae* (**a**)*, Mycosphaerellaceae* (**f**)*, Pleosporaceae* (**g**), *Fomitopsidaceae* (**h**)*,* and *unidentified_Branch02_sp* (**i**) were increased in MCT-PAH group, while *Sclerotiniaceae* (**j**) levels were lower. CON: control; MCT: monocrotaline; PAH: pulmonary arterial hypertension.

**Figure 5:**
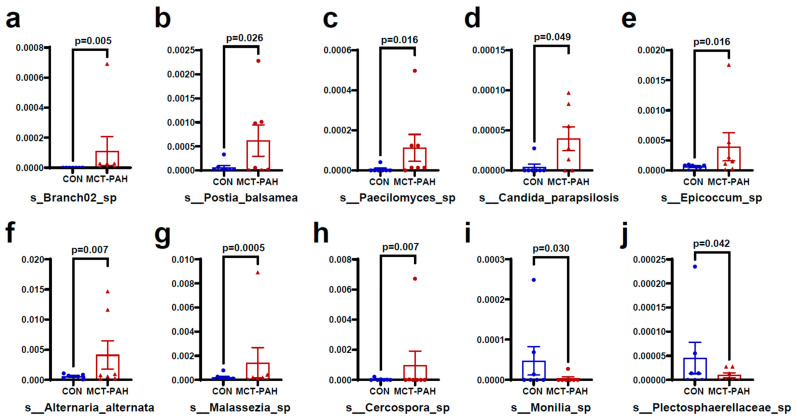
Fungal species differentially enriched in MCT-PAH group at the species level. At the species level, MCT rats showed higher levels of *Branch02_sp* (**a**), *Postia_balsamea* (**b**), *Paecilomyces_sp* (**c**), *Candida_parapsilosis* (**d**), *Epicoccum sp* (**e**), *Alternaria alternata* (**f**), *Malassezia sp* (**g**), and *Cercospora sp* (**h**), while *Monillia_sp* (**i**) *and Plectosphaerellaceae_sp* (**j**) were enriched in CON group. CON: control; MCT: monocrotaline; PAH: pulmonary arterial hypertension.

Figure 4 and Figure 5 present additional insights into the taxonomic changes observed at various classification levels. At the class level, there were increases in *Malasseziomycetes* and *Rozellomycotina_cls_Incertae_sedis* (Figure 4a,b)*,* as well as in the orders *Malasseziales, Branch02, Polyporales*, and *Capnodiales* (Figure 4a,c–e). At the family level, the MCT rats exhibited higher levels of *Malasseziaceae, Mycosphaerellaceae, Pleosporaceae, Fomitopsidaceae,* and *unidentified_Branch02_sp*, while *Sclerotiniaceae* levels were lower (Figure 4a,f–j). Furthermore, at the species level, MCT rats showed higher levels of *Branch02_sp, Postia_balsamea, Paecilomyces_sp, Candida_parapsilosis, Epicoccum sp, Alternaria alternata, Malassezia sp, Cercospora sp, Monillia_sp,* and *Plectosphaerellaceae_sp* (Figure 5a–j).

### 3.5. Interaction of Bacteria and Fungi in Pulmonary Arterial Hypertension

**Figure 6:**
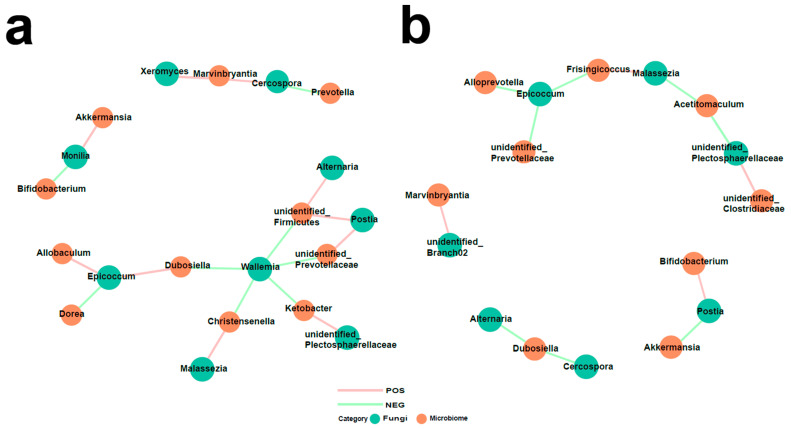
Interactions between differentially abundant gut fungi and gut bacteria in the CON and MCT-PAH groups. Inter-kingdom interactions between differentially abundant enteric fungi at the genus level and gut bacteria at the genus level in the CON group (**a**) and MCT-PAH group (**b**). The red and green colors indicate positive and negative correlations, respectively.

To investigate the relationships between fungi and bacteria in the gut, we also characterized the bacterial microbiome of the same samples using 16S rRNA (V3–V4) sequencing (Supplementary Figure S1). The observed shift in the bacteria in PAH rats in this study was consistent with previous reports [13]. *Firmicutes* emerged as the most abundant phylum, and the *Firmicutes* to *Bacteroidetes* ratio, considered a hallmark of gut dysbiosis, was also found to be increased in the PAH group. Subsequently, we identified several bacteria that were significantly altered and elucidated the interaction between the gut fungal and bacterial communities in the CON group and MCT-PAH group (Figure 6). A total of 18 and 12 inter-kingdom correlations were observed in the CON and MCT-PAH groups, respectively. The fungus *Malassezia* exhibited a positive correlation with the *bacterium Christensenella*, while the fungus *Wallemia* showed negative correlations with the bacteria *unidentified_Prevotellaceae, unidentified_Firmicutes, Ketobacter, Christensenella*, and *Dubosiella*. However, these correlations were no longer evident in the context of PAH.

## 4. Discussion

In the present work, we reveal, for the first time, a complex interplay between the gut mycobiome and pulmonary arterial hypertension in a MCT-induced rat model. Firstly, through ITS1 region sequencing, a diverse range of fecal fungi was identified, with no significant differences in alpha- and beta-diversity observed between the control and PAH rats. Notably, distinct genera such as *Malassezia* and *Epicoccum* were found to be significantly more abundant in the PAH group. Secondly, specific fungal genera exhibited significant correlations with hemodynamic indices, suggesting a potential link between gut fungi and PAH pathophysiology. Lastly, the study observed altered inter-kingdom correlations between gut fungi and bacteria in PAH, indicating a disrupted gut ecosystem in diseased states. These findings highlight the intricate interactions between the gut mycobiome and PAH, providing valuable insights into the potential role of gut fungi in the development and progression of this condition.

PAH is a pernicious cardiovascular disorder in which the pathogenesis and associated complications remain predominantly enigmatic.

Nonetheless, the role of gut microbiota and its metabolites in cardiovascular diseases has been extensively reported [2,14,15]. For PAH, a growing corpus of research implicates gut microbiota, particularly bacteria, as significant contributors to the pathophysiological landscape of PAH [16]. The gut microbiota encompasses a diverse consortium of organisms, including bacteria, viruses, fungi, and archaea. Notably, fungi represent a modest fraction, less than 1%, of the human gut microbiome as revealed by metagenomic sequencing [4]; yet, they are pivotal in preserving the equilibrium and functional integrity of the microbial community [17]. Perturbations in the gut mycobiota, or dysbiosis, have been linked to heightened susceptibility to a spectrum of maladies, such as diabetes, hepatocellular carcinoma, inflammatory bowel disease (IBD), and hypertension [8,9,11,18]. This investigation provides the inaugural evidence of gut fungal composition disruption in patients with PAH.

No significant difference in global mycobiome diversity was observed between the PAH and control groups. Alpha- and beta-diversity assessments typically depend on evaluating the dissimilarities between microbial communities based on the totality of microbial attributes. Yet, occasionally, it is a subset of the community that is of paramount importance. These critical but limited members may not significantly impact the aggregate dissimilarity measures, leading to their potential oversight in comprehensive comparisons. Additionally, patterns of alpha- and beta-diversity may be distorted by the inherent sparsity of data, particularly when the analysis is restricted to less prevalent microbial species [19,20]. Therefore, alterations in the mycobiome composition of rats afflicted with PAH were further investigated across various taxonomic ranks. As expected, multiple differential species were identified across various taxonomic levels.

Our results indicate that *Malassezia* may serve as a promising biomarker for pulmonary arterial hypertension (PAH), as evidenced by its augmented levels in the gastrointestinal tract of PAH-afflicted rats and its positive association with right ventricular systolic pressure (RVSP). While *Malassezia* is a known predominant fungal constituent of the skin microbiota, its proliferation in the gut mucosa may be implicated in the progression of PAH [21]. *Malassezia* species are characterized by their proinflammatory biological activities, which are linked to the promotion of inflammation-driven diseases. Specifically, *M. restricta* has been correlated with inflammatory bowel diseases such as Crohn’s disease and ulcerative colitis [22]. In germ-free mice, the introduction of *M. restricta* exacerbated intestinal inflammation by activating Card9 signaling through the Dectin-2 receptor, thereby intensifying the severity of ulcerative colitis. Moreover, the oncogenic potential of *Malassezia* has been substantiated in various cancer types. It has been shown that *Malassezia* can trigger the mannose-binding lectin and complement-3 pathways, contributing to the advancement of pancreatic ductal adenocarcinoma and hepatic cancer [9,23]. In other cardiovascular diseases such as hypertension, *Malassezia* was not only enriched in the state of hypertension condition but also closely correlated with the immune homeostasis of hypertensive patients [11]. Given its notable impact on the inflammatory mechanisms associated with PAH, *Malassezia* is emerging as a significant pathogen of interest. To further delineate the functional role of *Malassezia,* additional research utilizing animal models and fecal microbiome transplants from PAH subjects is warranted. Regarding the genus of *Epicoccum*, although its contribution to the pathogenesis of pulmonary arterial hypertension (PAH) has not been definitively elucidated, several studies have reported a notable increase of *Epicoccum* in the gastrointestinal tract of fluoride-poisoned mice and patients with multiple sclerosis [24,25]. These findings provide compelling evidence for the potential involvement of *Epicoccum* in the pathophysiology of these diseases. Further investigations are warranted to unravel the mechanisms underlying the potential role of *Epicoccum* in PAH.

The study’s identification of the correlations between fungal genera and hemodynamic indices in PAH is a notable addition to the literature. These findings align with previous research by Liu et al. and Jie et al., which have explored the gut microbiome’s influence on cardiovascular diseases, demonstrating associations between microbial compositions and clinical markers [26,27]. However, what sets our study apart is its unique focus on the mycobiome, rather than the bacteriome, in relation to PAH. By specifically examining the role of fungi, we provide novel insights into the potential involvement of the mycobiome in this context.

The study’s exploration of inter-kingdom interactions within the gut microbiome in PAH provides valuable context to the growing recognition of the complex interplay between different microbial kingdoms in health and disease. This aligns with the work of researchers like Huang and Lee [28,29], who have emphasized the importance of fungal–bacterial interactions in the gut ecosystem and their potential implications for host health.

The findings, particularly the lack of significant changes in the overall fungal diversity and the specific alterations at the genus level, provide a nuanced perspective compared to previous studies. While some earlier research has focused on broad changes in the gut mycobiome in various diseases, our study underscores the importance of looking at specific taxonomic shifts and their potential impact on disease pathology.

This study significantly contributes to the understanding of PAH in several ways. Firstly, it expands our knowledge about the role of the gut–lung axis in PAH, particularly highlighting the significance of the gut mycobiome. Secondly, our findings about the specific fungal genera associated with PAH pathophysiology could pave the way for novel diagnostic and therapeutic strategies. Finally, the study underscores the importance of integrative approaches, combining hemodynamic assessments with microbiome analysis, in understanding complex diseases like PAH.

One limitation of this study is the absence of direct causal evidence that establishes a link between changes in the gut mycobiome and the development of PAH. Future research should aim to establish this causality, perhaps through interventional studies manipulating the gut mycobiome. Additionally, the precise mechanisms through which specific fungal taxa influence PAH remain unclear and warrant further investigation. Expanding this research to human subjects and incorporating a broader range of microbial analyses will be essential steps in translating these findings into clinical practice.

## 5. Conclusions

In summary, our findings provide pioneering insights into the role of the gut mycobiome in pulmonary arterial hypertension (PAH), particularly through the identification of specific fungal genera like *Malassezia* that are significantly more abundant in PAH and may serve as potential biomarkers. Despite no significant differences in overall fungal diversity, the distinct taxonomic shifts and their correlations with hemodynamic indices highlight the intricate relationship between the gut mycobiome and PAH. These findings emphasize the importance of the gut–lung axis and suggest that targeting the gut mycobiome could offer novel diagnostic and therapeutic avenues. However, the absence of direct causal evidence and unclear mechanisms of action calls for further research, including interventional studies and expansion to human subjects, to fully understand the implications of these microbial changes in PAH.

## Figures and Tables

**Table 1:** The fungal sequence information of each sample.

Sample	Raw Reads	Clean Reads	Effective Reads	AvgLen (bp)	GC (%)	Effective (%)
CON1	92,726	90,560	88,665	221	48.39	95.62
CON2	82,268	81,188	79,497	222	48.39	96.63
CON3	75,580	74,117	72,526	222	48.26	95.96
CON4	85,737	85,089	83,417	222	48.43	97.29
CON5	85,209	83,826	82,133	226	48.16	96.39
CON6	89,549	88,400	86,154	222	48.34	96.21
CON7	88,015	86,300	84,055	223	48.27	95.5
MCT1	89,709	89,113	87,205	223	48.43	97.21
MCT2	83,547	83,056	81,245	228	48.15	97.24
MCT3	79,204	77,489	75,775	224	48.24	95.67
MCT4	81,053	80,264	78,504	226	48.03	96.86
MCT5	82,838	82,001	80,003	221	48.21	96.58
MCT6	78,948	77,663	75,731	224	48.28	95.93
MCT7	90,372	88,565	86,191	223	48.37	95.37

Raw reads: the total number of reads generated from sequencing. Clean reads: the sequences have been filtered for low quality and short length after processing the raw reads. Effective reads: the sequences have been further filtered for chimeras and are ultimately used for subsequent analysis.

## Data Availability

Raw sequences were deposited in the NCBI Sequence Read Archive under accession number PRJNA975055.

## Supplementary Materials

The following supporting information can be downloaded at: https://www.mdpi.com/article/10.3390/biomedicines12020298/s1, Figure S1: Gut Bacterial Analysis in Control (CON) and MCT-PAH Groups.
