# Supplementary material for: Gut Fungal Microbiota Alterations in Pulmonary Arterial Hypertensive Rats
Source: Biomedicines. 2024 Jan 27;12(2):298. doi: 10.3390/biomedicines12020298 (PMC10886911; doi:10.3390/biomedicines12020298)
Supplement: Supplementary file 1 [file biomedicines-12-00298-s001.zip › biomedicines-2781858-supplementary.pdf]

Supplementary Material

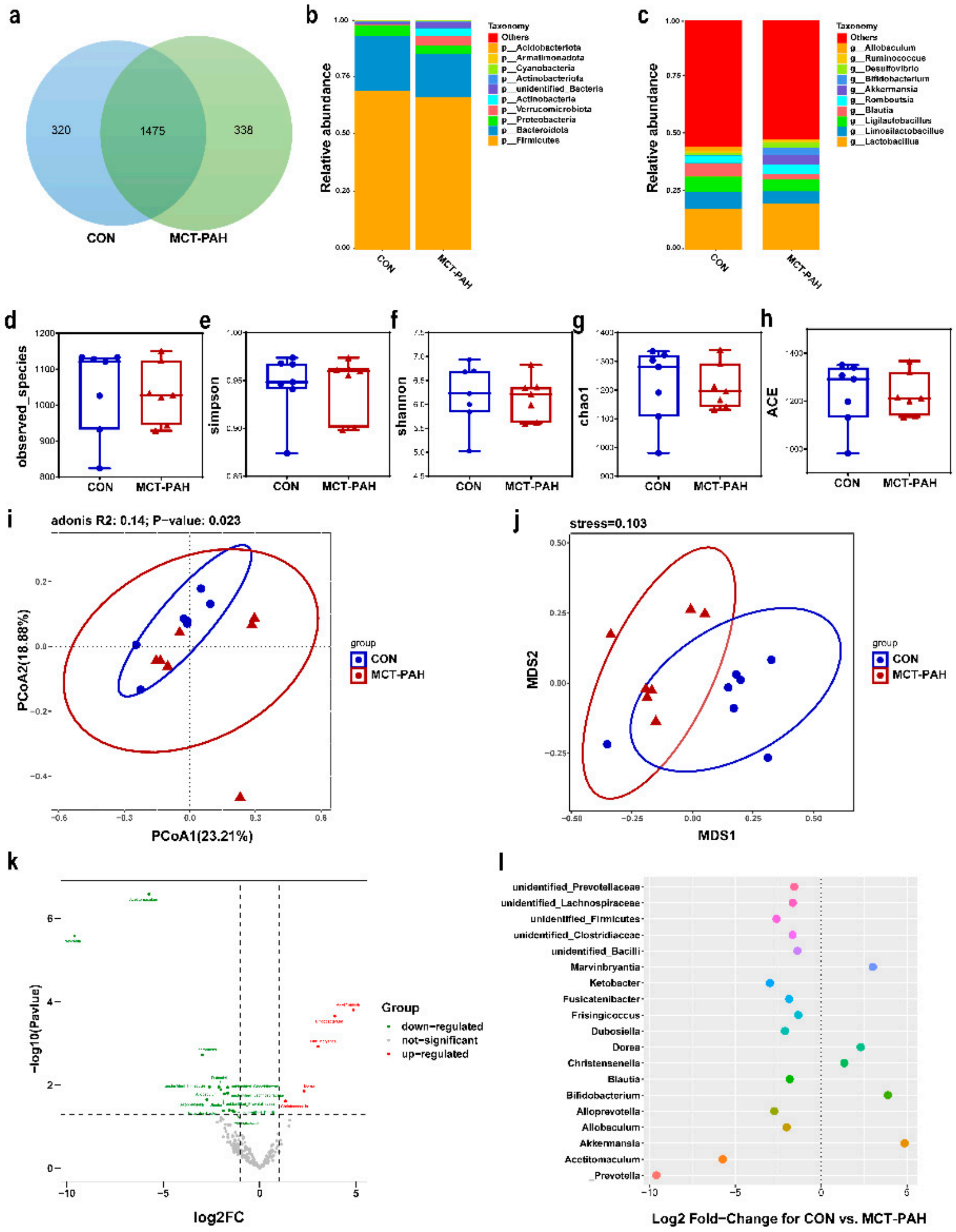

Supplementary Figure S1. Gut Bacterial Analysis in Control (CON) and MCT-PAH Groups. (a) Venn diagrams illustrating the distribution of gut bacterial operational taxonomic units (OTUs). (b-c) Comparative analysis of the relative abundance and composition of gut bacteria at the phylum and genus levels in CON and MCT-PAH groups. (d-h) Statistical analysis of alpha diversity between the two groups, including (d) observed species index, (e) Simpson index, (f) Shannon index, (g) Chao1 index, and (h) ACE index. No significance is denoted as "ns". (i) Principal coordinates analysis (PCoA) diagram based on Bray-Curtis dissimilarity; (j) Non-metric multidimensional scaling (NMDS) plots based on Bray-Curtis dissimilarity. (k) Volcano plot depicting differential abundance of bacterial species at the genus level between CON and MCT-PAH groups. (l) Dot plot representation of significantly differentially abundant bacterial species in the PAH group. Statistical significance was assessed using the PERMANOVA test. MCT: monocrotaline. PAH: pulmonary arterial hypertension.
